# Natriuretic Peptides: It Is Time for Guided Therapeutic Strategies Based on Their Molecular Mechanisms

**DOI:** 10.3390/ijms24065131

**Published:** 2023-03-07

**Authors:** Giovanna Gallo, Speranza Rubattu, Camillo Autore, Massimo Volpe

**Affiliations:** 1 Department of Clinical and Molecular Medicine, Sapienza University of Rome, Sant’Andrea Hospital, Via di Grottarossa 1035, 00189 Rome, RM, Italy; 2 IRCCS Neuromed, Via Atinense 18, 86077 Pozzilli, IS, Italy; 3 IRCCS San Raffaele Cassino, Via G. Di Biasio 1, 03043 Cassino, FR, Italy; 4 IRCCS San Raffaele Roma, Via della Pisana 235, 00163 Rome, RM, Italy

**Keywords:** natriuretic peptides, heart failure, cardiovascular diseases

## Abstract

Natriuretic peptides (NPs) are the principal expression products of the endocrine function of the heart. They exert several beneficial effects, mostly mediated through guanylate cyclase-A coupled receptors, including natriuresis, diuresis, vasorelaxation, blood volume and blood pressure reduction, and regulation of electrolyte homeostasis. As a result of their biological functions, NPs counterbalance neurohormonal dysregulation in heart failure and other cardiovascular diseases. NPs have been also validated as diagnostic and prognostic biomarkers in cardiovascular diseases such as atrial fibrillation, coronary artery disease, and valvular heart disease, as well as in the presence of left ventricular hypertrophy and severe cardiac remodeling. Serial measurements of their levels may be used to contribute to more accurate risk stratification by identifying patients who are more likely to experience death from cardiovascular causes, heart failure, and cardiac hospitalizations and to guide tailored pharmacological and non-pharmacological strategies with the aim to improve clinical outcomes. On these premises, multiple therapeutic strategies based on the biological properties of NPs have been attempted to develop new targeted cardiovascular therapies. Apart from the introduction of the class of angiotensin receptor/neprilysin inhibitors to the current management of heart failure, novel promising molecules including M-atrial natriuretic peptide (a novel atrial NP-based compound) have been tested for the treatment of human hypertension with promising results. Moreover, different therapeutic strategies based on the molecular mechanisms involved in NP regulation and function are under development for the management of heart failure, hypertension, and other cardiovascular conditions.

## 1. Regulation of Natriuretic Peptide Synthesis and Secretion

Natriuretic peptides (NPs) are the principal expression products of the hormonal function of the heart.

The main components of the NP family are atrial natriuretic peptides (ANPs), brain or B-type natriuretic peptide (BNPs), and C-type natriuretic peptides (CNPs). ANPs and BNPs are mostly synthesized in the heart whereas CNPs are produced by endothelial cells [1,2,3,4]. 

NPs exert relevant physiological cardiovascular and renal effects, which include natriuresis, diuresis, vasorelaxation, blood volume and blood pressure reduction, trans-capillary fluid shift, increase in glomerular filtration rate, and inhibition of the renin-angiotensin-aldosterone system (RAAS) [1,2,3,4]. 

Apart from the endocrine functions, NPs exert several autocrine/paracrine effects, including lipolysis, reduction in inflammation and of oxidative stress, stimulation of autophagy, and anti-fibrotic and anti-hypertrophic actions which play a role in cardiovascular pathophysiology [5,6]. 

The production of ANPs and BNPs by cardiomyocytes occurs mostly as a consequence of pressure and volume overload and entails regulated secretion deriving from stored granules and a constitutive secretion of newly synthesized hormones [7,8,9,10]. At least three distinct mechanisms are involved in the stimulated secretion of NPs: stretch-activated Goα-coupled secretion, Gqα-coupled secretion, and cytokine-promoted secretion. Acute mechanical stretch, which occurs within hours, produces a short-term burst of regulated secretion of NPs, without evident effects on their biosynthesis. In subacute (days) and chronic (weeks) haemodynamic load conditions circulating NPs derive from both stored and newly synthesized molecule [7,8,9,10]. 

In fact, the prohormones proANP and proBNP are stored in secretory granules, are enzymatically cleaved by proprotein convertases (corin and furin), and are processed to form the N-terminal peptides NT-proANP and NT-proBNP and the biologically active COOH-terminal peptides ANP and BNP [11]. The main circulating form of ANP is the COOH-terminal peptide (αANP), while BNPs and NT-proBNPs circulate in the plasma along with unprocessed proBNPs and other inactive fragments [12,13]. In normal conditions, ANPs and BNPs are mostly produced within the atria and, more specifically, within the auricular appendage [14]. Studies performed with electron microscopy have shown that secretory granules are not displayed in ventricular cardiomyocytes from normal hearts, but they may be detected in samples of ventricular myocardium collected during surgery or endocardial biopsies in patients with cardiac disease [15]. These data suggest that NP synthesis and secretion are differently regulated in atrial cardiomyocytes as compared to ventricular cardiomyocytes and that pathological conditions leading to increased wall stress stimulate the pre-programmed expression of fetal genes in ventricular cardiomyocytes [15]. It has been also demonstrated that additional ventricular BNP expression occurs in clinically overt HF, suggesting that in this condition the atrial secretion might not be sufficient to counteract the activation of vasoconstrictive and sodium retaining neurohormonal systems, such as the renin-angiotensin-aldosterone and sympathetic nervous systems [16]. These findings were confirmed in an advanced HF setting of heart transplantation, further supporting the hypothesis that ventricular production mostly occurs under advanced pathological conditions and is related to ventricular remodeling and other abnormalities associated with hypertrophy, fibrosis, inflammation, and hypoxia [17,18]. 

Besides the mechanical triggering stimulus occurring in conditions of volume and pressure overload, different neurohormonal stimuli, including angiotensin II, endothelin-1, arginine vasopressin, adrenergic agonists, growth factors and cytokines (tumor necrosis factor and interleukins −1 and 6), glucocorticoids, thyroid hormones, sex steroids, and the incretin glucagon-like peptide-1 (GLP1) [19,20], may promote increased secretion of NPs. The neurohormonal stimuli regulate the expression of both *NPPA* and *NPPB*, the genes encoding ANP and BNP, respectively, through the transcriptional factors RAS/c-Raf-1 and the phosphoinositide signaling pathways which stimulate the p38-mitogen-activated protein kinase (MAPK) [21,22,23]. 

**Figure 1:**
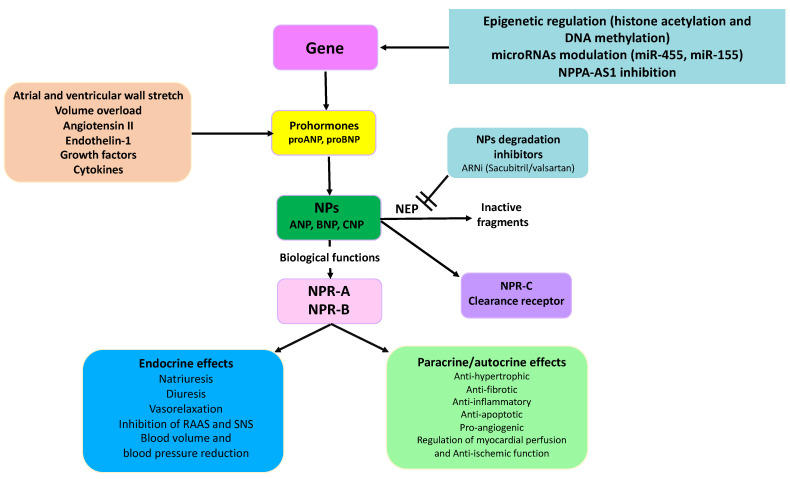
Schematic representation of the natriuretic peptide system from secretion to biological actions. Legend to Figure 1. NPs are produced by cardiomyocytes upon hemodynamic and neuroendocrine stimuli. Cardiomyocytes store prohormones, namely proANP and proBNP, that are then processed to the biologically active peptides ANP and BNP. NPs act on target cells through specific cGMP-coupled receptors.

Epigenetic regulation, consisting in histone acetylation and methylation, also plays a role in the synthesis of NPs [24]. Pressure and volume overload have been related to the enhanced acetylation of H3 and H4 histones and to the demethylation of H3K9 at *NPPA* and *NPPB*. In these conditions, the expression of three histone methylation-related molecules H3K4me3, H3K9me2, and H3K9me3 is reduced [25,26,27]. The *NPPA* expression is also modulated by known miRNAs, such as miR-425, miR-155, and miR-105 [28,29,30]. Indeed, subjects carrying the minor G allele at rs5068, which does not bind miR-425, show higher ANP levels resulting in a lower risk of CVD, obesity, and hypertension [31,32,33]. Moreover, the inhibition of the NPPA-AS1 natural antisense transcript, which reduces the *NPPA* transcription, increases both ANP levels and cGMP resulting in BP reduction [34] (Figure 1).

The orange boxes represent the regulatory mechanisms of gene expression and the secretion of NPs, including new potential strategies such as epigenetic regulation, microRNA modulation, and *NPPA* AS1 inhibition. 

The light-blue boxes illustrate NP-based potential therapeutic strategies. 

ANP, atrial natriuretic peptide; ARNi, angiotensin receptor/neprilysin inhibitors; BNP, brain natriuretic peptide; CNP, C-type natriuretic peptide; miR, microRNA; NEP, neprilysin; NPR-A, type A natriuretic peptide receptor; NPR-B, type B natriuretic peptide receptor; NPR-C, type C natriuretic peptide receptor; NPs, natriuretic peptides

## 2. Natriuretic Peptide Receptors

The biological actions of NPs are mediated by three receptors: type A (NPR-A), type B (NPR-B), and type C (NPR-C) NP receptors [35]. NPR-A is the main receptor for ANP and BNP, with ANP showing the greatest affinity to NPR-A, followed by BNP and CNP [35]. CNP has the highest affinity to NPR-B, followed by ANP and BNP. BNP has the highest affinity to NPR-C, followed by CNP and ANP. NPR-A and NPR-B are expressed in many tissues, including the heart, vessels, kidneys, brain, lungs, adrenal glands, and adipose tissue. NPR-A and NPR-B are composed of three domains: an extracellular domain that binds NPs, a short transmembrane domain, an intracellular domain that contains a protein kinase homology domain, a helical hinge region, and a guanylyl cyclase domain [36]. In the absence of stimuli, the protein kinase domain of NPR-A is phosphorylated, whereas when a ligand binding occurs, phosphates are removed resulting in receptor desensitization. The hinge region is involved in the oligomerization of NP receptors and in the activation of the catalytic domain [36]. 

Differently from NPR-A and NPR-B, NPR-C is characterized by a cell membrane dimeric conformation and does not have guanylyl cyclase activity. NPR-C internalizes NPs leading to lysosome degradation and to their clearance [37]. Neprilysin is the other major catalytic pathway involved in the degradation of NPs and other biologically active peptides, including bradykinins, substance P, enkephalins, and adrenomedullin [38].

The NPs binding to NPR-A and NPR-B cause the intracellular formation of cyclic guanylate monophosphate (cGMP) whose intracellular targets are cGMP-dependent protein kinases (PKGs), cGMP-gated ion channels, and cGMP-regulated cyclic nucleotide phosphodiesterases [39,40]. 

PKG phosphorylates Ca2+-activated K+ channels and inositol trisphosphate receptor-associated cGMP kinase substrates, resulting in a reduction in the cytosolic concentration of Ca2+ and hence the relaxation of vascular smooth muscle cells [41]. In addition to these biological mechanisms, endothelium derived CNP contributes to maintaining endothelial function and integrity and possibly to the chronic regulation of vascular tone, promoting vasorelaxation in isolated resistance arteries and BP reduction [42,43]. PKG1α plays an important role in the inhibition of inflammation and leukocyte recruitment, platelet aggregation, smooth muscle proliferation, vasoconstriction, fibrosis, coronary microvascular impairment, and hypertrophy through different downstream mechanisms, including SR Ca2+-ATPase (SERCA)-mediated increased re-uptake of Ca2+ into the sarcoplasmic reticulum and the blockade of mitochondrial permeability transition pore [44,45].

NPR-B also plays a role in maintaining normal heart rate and sino-atrial node function by modulating ion channel function via a cGMP/phosphodiesterase-3 (PDE3)/cyclic adenosine monophosphate (cAMP) signaling mechanism [46].

**Figure 2:**
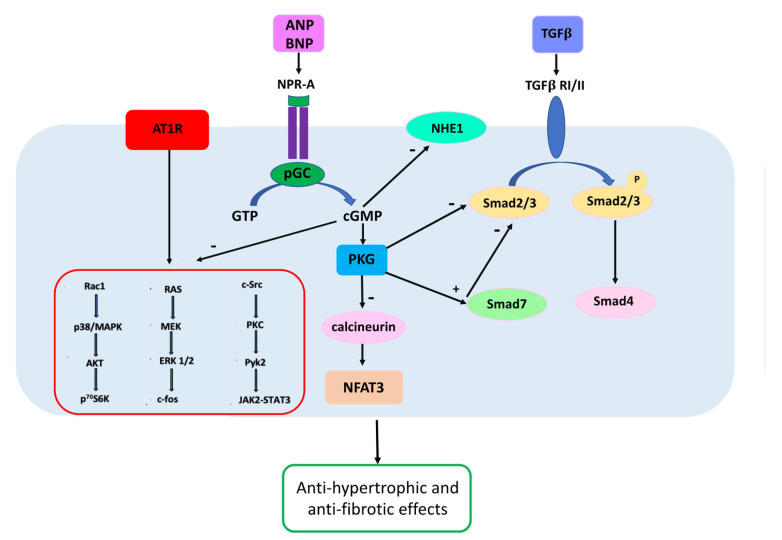
Anti-hypertrophic and anti-fibrotic effects of natriuretic peptides. Legend to Figure 2: ANPs and BNPs antagonize calcineurin/NFAT, NHE-1, TGF-β1, and angiotensin II signaling. ANP, atrial natriuretic peptide; cGMP, cyclic guanosine monophosphate; ERK, extracellular-signal-regulated kinase; JAK, Janus kinase; JNK, c-JUN N-terminal kinase; MAPK, p38 mitogen-activated protein kinase; MEK, mitogen-activated protein kinase; NFAT, nuclear factor of activated T cells; NHE, Na+/H+ exchanger; NPR-A, type A natriuretic peptide receptor; NP, natriuretic peptide; p^70^S6K, ribosomal protein S6 kinase beta-1; PKG, protein kinase G; PyK2, proline-rich tyrosine kinase 2; Rac1, Ras-related C3 botulinum toxin substrate; Smad, suppressor of mothers against decapentaplegic; STAT3 signal transducer and activator of transcription 3; TGF, tumor growth factor.

NPs exert anti-hypertrophic and anti-fibrotic effects through the modulation of several pathways: calcineurin/NFAT (nuclear factor of activated T-cells), the sodium exchanger NHE-1 (sodium-hydrogen antiporter 1), and the TGFβ1 (transforming growth factor β1)/Smad signaling pathways. NPs reduce vascular inflammation by inhibiting the TNFα-induced expression of adhesion molecules, such as E-selectin and ICAM-1. NPs also inhibit the pathway of RAC1 (Ras-related C3 botulinum toxin substrate)/p38/MAPK (mitogen activated protein kinase), which is involved in the activation of Akt and mTOR and its downstream target p^70^S6K, the Ras/MEK (mitogen-activated protein kinase)/ERK (extracellular-signal-regulated kinase) signaling. These biological pathways lead to an increased expression of c-fos mRNA, JNK (c-JUN N-terminal kinase), and c-Src, which stimulate JAK2 (Janus kinase 2)-STAT3 (signal transducer and activator of transcription 3) through PKC (protein kinase C) and PyK2 (proline-rich tyrosine kinase 2) [47,48,49] (Figure 2).

Consistently, the ablation of *NPPA* or *NPR1* (the gene encoding NPR-A) as well as NP genetic variants leading to reduced peptide expression have been associated with cardiac hypertrophy and impaired endothelial cell viability and proliferation [50,51,52,53,54]. A long-term proBNP peptide gene delivery exerted an anti-hypertrophic effect and improved both systolic and diastolic function in the spontaneously hypertensive rat [55]. An *NPPA* mutation was associated with the enhanced expression and function of a cardiac potassium channel and to mitochondrial electron transport chain dysfunction, resulting in an electrophysiological substrate for atrial fibrillation [56,57]. Moreover, ANPs were recently discovered to regulate cardiac autophagy, reducing the cellular quote of damaged cells and organelles, and finally protecting the heart from ischemic insult [58]. 

Resistance to the biological effects of NPs can be attributed to different mechanisms acting at pre-receptor, receptor, and post-receptor levels. The impaired post-translational processing of proANP and proBNP was associated with NP resistance in HF, as well as the reduced corin and furin levels and the glycosylation of proBNP near the cleavage site of the enzyme, resulting in the resistance to proteolysis [59]. An increased expression of the clearance receptor NPR-C was associated with a reduction in the circulating levels of active NPs. Different gene polymorphisms of NP receptors may contribute to the development of CVD [60]. Moreover, the increased metabolism of cGMP due to the enhanced activity of PDE may also contribute to the increased resistance of NPs. Although the substitution of exogenous NO by nitrates and the inhibition of cGMP degradation by PDE inhibitors have shown some potential benefits in the treatment of HF, a long-term effect has not been demonstrated [61].

## 3. The Key Role of Natriuretic Peptides as Clinical Biomarkers in Cardiovascular Diseases

BNP and NT-proBNP represent widely recommended biomarkers for the diagnosis of acute decompensated HF and also have an important prognostic role since their increase tightly reflect HF worsening and predict outcomes in HF patients. According to current international guidelines, cut-off values for BNP and NT-proBNP are in routine use for ruling in or ruling out HF [62], since they represent a more stable and reproducible parameter in the clinical work-up.

The role of NPs as biomarkers to guide the clinical behavior in HF has resulted in an improvement in clinical outcomes, though they did not specifically address the impact on the intensity of care. In fact, in the recent COACH (Comparison of Outcomes and Access to Care for Heart Failure) trial, the use of a point-of-care tool for risk stratification in the emergency department to support clinical decision-making and rapid outpatient follow-up was associated with an improvement of 30-day and 20-month clinical outcomes [63]. In this approach, the use of NPs as routine biomarkers most likely played a role [64]. In addition to BNPs and NT-proBNPs, different studies have shown that ANPs and MR-proANPs may representuseful tools for the diagnosis of HF and are associated with increased all-cause and cardiovascular mortality, although their diagnostic values as biomarkers have not been extensively investigated [1,65]. In HF patients enrolled in the Atherosclerosis Risk in Community study, NT-proBNP level >125 pg/mL reflected an increased risk for HF events and death, supporting the utility of serial NT-proBNP measurements to improve risk stratification [66].

In addition to being well-established markers of HF, plasma levels of NPs are also risk markers for different CVD such as atrial fibrillation (AF), coronary artery disease, and valvular heart disease, as well as in the presence of left ventricular hypertrophy [67,68] and severe cardiac remodeling [69]. Higher levels of BNPs and NT-proBNPs were detected in subjects with grade-2 hypertension compared to normotensives, corresponding to an increased risk of organ damage and hypertension-related sequelae [70]. On the other hand, a deficiency of NPs was reported in the early stage of hypertension, and it was proposed as a contributory pathogenetic mechanism [71,72]. NP levels are increased in >75% of patients with AF and were associated with an increased risk of stroke, systemic embolism, and cardiac-related death, thus enhancing the prognostic role of the CHA2DS2-VASc score [73]. NPs are sensitive markers of both clinically evident and subclinical myocardial ischemia, being significantly more expressed in human coronary explants of advanced atherosclerotic lesions compared to early atherosclerotic lesions [74]. NP levels were able to predict future cardiovascular events in subjects with previous myocardial infarction [75]. In a 10-year follow-up study performed in patients with stable coronary artery disease, NT-proBNP levels within the highest quartile were associated with a 50% survival rate and with an increased risk of myocardial infarction cardiovascular, and all-cause death [76]. 

In valvular heart disease, NPs associated with the severity of aortic stenosis, LV chamber size, wall thickness, wall stress, LVEF, left atrial size, and right ventricular pressure are useful markers for monitoring the evolution from asymptomatic to symptomatic disease and finally to HF [77]. In mitral regurgitation NPs contribute to assess the progression of the valve disease, even before the development of hemodynamic consequences in a preclinical stage of myocardial damage. They may also contribute to more accurate risk stratification by identifying patients who are more likely to experience death from cardiovascular causes, HF, and cardiac hospitalizations, thus requiring surgical management rather than a conservative approach [78].

In patients with pulmonary arterial hypertension, BNP levels showed a direct correlation with the mean pulmonary artery pressure, namely with a substantial increase in mortality when accompanied by an enlarged right atrium measured by echocardiography [79,80,81,82]. NPs predict mortality in patients with end-stage renal disease [83].

## 4. Therapeutic Implications of the Regulation of Natriuretic Peptide Levels and Activity

Over the last few years, the regulation of NP levels and activity have established broad applications in clinical practice due to the development of a new therapeutic strategy, namely the angiotensin receptor neprilysin inhibitor (ARNi): the single pill sacubitril/valsartan aimed at combining the effect of reduced enzymatic degradation of NPs with consequent increased NP levels and reduced angiotensin II receptor binding and related signal transduction pathways. Due to the higher affinity of neprilysin for ANP and CNP than for BNP, neprilysin inhibition increases the ANP carboxyterminal level. The parallel decrease of the circulating amino-terminal peptides observed upon ARNi treatment mirrors the cardiac function improvement [38]. 

In the proof-of-concept study PARADIGM-HF, which enrolled 8442 patients with HFrEF (EF ≤ 35%, ≤40% before the amendment) and NYHA class II to IV, sacubitril/valsartan reduced the risk of the composite primary outcome of cardiovascular death or hospitalization for HF by 20% compared with enalapril. Sacubitril/valsartan also lowered the risk of cardiovascular death by 20% and HF hospitalization by 21% compared to enalapril [84]. The benefits of sacubitril/valsartan were independent from LVEF, age, renal function, BP, HF etiology (ischemic, idiopathic, hypertensive, or other non-ischemic causes such as infective/viral, alcoholic, valvular, drug-related, and peripartum-related), previous hospitalizations, signs of congestion, background pharmacological therapy, glycemic status, and geographic origin [84]. The improvement of cardiovascular outcomes in the group treated with sacubitril/valsartan was mirrored by a reduction in the NT-proBNP level. In the group who received sacubitril/valsartan, the BNP level increased at 30 days after randomization but tended to be lower at the 8-month follow-up compared to the group treated with enalapril. Otherwise, the NT-proBNP levels were reduced at 30 days and 8 months in the active group [85,86]. 

The efficacy of sacubitril/valsartan was also demonstrated in acute subsets. The TITRATION study showed that initiation of sacubitril/valsartan and subsequent up-titration in both hospitalized and outpatient HFrEF patients with LVEF ≤ 35% had a similar tolerability profile without significant differences in the occurrence of adverse events such as hyperkalemia, angioedema, renal dysfunction, or hypotension [87]. In the PIONEER-HF study conducted in patients hospitalized for acute HF, treatment with sacubitril/valsartan produced a 29% greater reduction in the NT-proBNP plasma concentration compared with enalapril (percent change, −46.7% vs. −25.3%), without significant differences in the risk of adverse events. Moreover, patients treated with sacubitril/valsartan experienced a lower incidence of all-cause mortality, rehospitalization for HF, left ventricular assist device implantation, or listing for cardiac transplant and of the composite of cardiovascular death or rehospitalization for HF [88]. In the TRANSITION trial, the target dose of sacubitril 97 mg and valsartan 103 mg twice daily after 10 weeks was achieved in a comparable percentage of hospitalized patients who started the treatment ≥12-h pre-discharge or between days 1–14 post-discharge, with a similar incidence of discontinuation due to adverse events [89].

In the PARAGON-HF trial, the efficacy of sacubitril/valsartan compared to valsartan alone was investigated in 4822 patients with LVEF of 45% or higher. Although a trend towards a reduction in the composite endpoint of cardiovascular death and total hospitalizations for HF was observed in the sacubitril/valsartan arm compared to valsartan (Rate ratio 0.87; 95%CI 0.75–1.01, *p* = 0.06), statistical significance was not reached [90]. 

More recently, Solomon and colleagues performed a pooled analysis of these two trials with the aim to evaluate the efficacy of sacubitril/valsartan across the EF spectrum. In this cohort of >13,000 subjects, sacubitril/valsartan was associated with a significant reduction in the combined endpoint of cardiovascular mortality and first hospitalization for HF (−16%), in all-cause mortality (−12%), total HF hospitalizations and cardiovascular mortality (−18%), and total HF hospitalizations (−19%) [91]. 

On the basis of this evidence, the European Guidelines recommend sacubitril/valsartan as first-line treatment in HFrEF and suggest considering its use in HF with mildly reduced EF (40–49%) [62,92,93]. 

In the PARADISE-MI (Prospective ARNI vs. ACE Inhibitor Trial to Determine Superiority in Reducing Heart Failure Events after Myocardial Infarction) study, sacubitril/valsartan did not produce significant benefits compared to ramipril on the composite outcome of cardiovascular death, HF hospitalizations, or outpatient development of HF in patients with acute myocardial infarction [94]. However, a superiority of sacubitril/valsartan was reported in a recent posthoc analysis of this study including only investigator-identified events [95]. 

Different studies have hypothesized and proven that the benefits of sacubitril/valsartan might be related to reverse cardiac remodeling. In the PROVE-HF study, the reduction in NT-proBNP levels was associated with the improvement of cardiac systolic and diastolic function consisting in the increase in LVEF and in the decrease of LV end-diastolic volume index (LVEDVI), LV end-systolic volume index (LVESVI), left atrial volume index (LAVI), and early diastolic filling–early diastolic annular velocity (E/eʹ) [96]. In the EVALUATE-HF study, sacubitril/valsartan reduced the remodeling indices (LVEDVI, LVESVI, LAVI, and E/eʹ) compared to enalapril, although without significant effects on LVEF, longitudinal strain, or aortic characteristic impedance [97]. 

An anti-fibrotic effect of sacubitril/valsartan was also demonstrated, with a reduction in the levels of different markers of tissue fibrosis such as ST2, tissue inhibitor of metalloproteinases-1 (TIMP-1), N-terminal pro-peptide of type I procollagen, and matrix metalloproteinases. In this context, the reduction in TIMP-1 levels is associated with a lower risk of cardiovascular events [98]. 

This large body of data may support the role of serial measurements of NP levels to guide the titration of long-term medical therapy in HF patients with the aim of reducing HF hospitalizations and improving clinical outcomes. NP activity might be enhanced by their interaction with other post-receptor pathways. In experimental models of HF, soluble guanylate cyclase (sGC) stimulators normalized endothelial function and improved sensitivity to NO, pulmonary arterial pressure, and pulmonary capillary wedge pressure, producing an increase in cardiac output [99]. Both stimulation and activation were developed as mechanisms directly acting on sGC. Soluble GC stimulation may occur by sensitizing sGC to lower levels of NO by stabilizing the nitrosyl-heme complex, which results in a long-lasting active configuration of the enzyme, or by increasing sGC activity through NO-independent binding to the enzyme [100,101]. Vericiguat, a sGC stimulator, was developed and tested in HfrEF. The VICTORIA (the Vericiguat Global Study in Subjects with Heart Failure with Reduced Ejection Fraction) study, a phase 3, randomized, double-blind, placebo-controlled trial, investigated the effects of vericiguat on top of standard therapy (target dose 10 mg) in a population of 5050 patients with worsening HF, LVEF < 45%, and New York Heart Association class II–IV, who experienced hospitalization for HF within 6 months before randomization or needed intravenous diuretic therapy, without hospitalization, within the previous 3 months 93. Compared to the placebo, vericiguat significantly reduced the primary composite outcome of death from cardiovascular causes or first hospitalization for HF (−10%), the secondary endpoints of total hospitalizations for HF (−9%), and death from any cause or first hospitalization for HF (−10%) [102]. The enhancement of cardiovascular protective effects of NPs linked to cGMP activation could be enhanced in the long term with sGC stimulation although this needs to be tested and proven [96]. Based on these results, vericiguat was introduced as a therapeutic tool for resistant HF in the most recent European guidelines on HF management [62]. 

## 5. Novel NP-Based Therapeutic Strategies

Besides ARNi, different NP based therapeutic strategies were developed in the last few decades [103,104]. In the 1990s, recombinant human ANP (carperitide) and synthetic human BNP (nesiritide) were approved in Japan and the United States, respectively, for intravenous infusion in acute decompensated HF [105]. However, the phase III ASCEND-HF trial did not demonstrate significant benefits on cardiovascular outcomes in patients treated with nesiritide who otherwise developed severe hypotension [106]. Based on these results, the Food and Drug Administration recommended the discontinuation of the manufacture of nesiritide in 2018.

Cenderitide, designed as CNP added to the C-terminal tail of dendroaspis natriuretic peptide (DNP), was designed to stimulate both NPR-A and NPR-B and is under investigation to evaluate its effects on natriuresis, diuresis, cardiovascular unloading, and cardiac remodeling [107]. 

More recently, mutant-ANP (MANP) was shown to stimulate cGMP activity in human cardiomyocytes and endothelial cells and to produce sustained BP reduction in animal models and in the first human study without significant adverse effects. MANP is more resistant to neprilysin and insulin-induced proteolytic degradation compared to ANP, therefore showing more prolonged diuretic, natriuretic, and antihypertensive effects [108,109,110]. MANP also inhibits aldosterone synthesis via PDE2 by a reduction in intracellular Ca2+ levels [111].

Another fragment derived from NT-proANP, the proANP 31–67 peptide, has shown cardiorenal protective actions in a preclinical model of hypertension-mediated organ damage with cardiac hypertrophy and renal damage [112]. 

The therapeutic efficacy of BNP was proven in the context of experimental congestive heart failure where the coadministration of BNP and furosemide maximized natriuretic and diuretic responses, preserved renal function, and inhibited aldosterone activation [113]. Therefore, several attempts were performed to obtain peptides derived from BNP to be used in clinical practice. One of them, called CRRL269, is characterized by the ring structure of BNP and the C-terminal and N-terminal tails of urodilatin, one of the precursors of ANP, as well as an additional four amino acids on the N-terminal end. In cellular models, CRRL269 demonstrated a cGMP production equivalent to BNP and urodilatin in HEK cells overexpressing NPR-A [114]. NPA7, a peptide that replaces the N-terminal tail of BNP with Ang1–7 combining the stimulation of NPR-A and Mas receptors, is currently under investigation [115]. 

Additional designer peptides are represented by ASBNP.1, derived from an alternative spliced transcript form of BNP [116] and by the conjugated ANP consisting of ANP-Fc (ANP fused to the Fc domain of IgG), ANP-HAS (recombinant human ANP and human serum albumin), and CNP/ANP [117].

Sangaralingham and colleagues have recently identified the NPR-A(GC-A) positive allosteric modulator (PAM) MCUF-651 which could enhance the binding affinity of both ANP and BNP to the receptor with a dose-dependent augmentation of cGMP production [118]. 

Finally, innovative strategies are under development with the aim to increase the circulating NP level through genetic/epigenetic modulation and, particularly, through RNA-based targets including miR-425 and miR-155 [28,29,30]. 

Future studies conducted in large patient cohorts with long follow-ups will validate these novel compounds for the prevention and clinical management of hypertension, its related target organ damage, and HF. Moreover, the potential efficacy and safety of these molecules need to be investigated in combination with other drugs currently used in clinical practice.

## 6. Conclusions

NPs exert different endocrine effects on both cardiovascular and renal systems counterbalancing the overactivation of the renin-angiotensin-aldosterone and sympathetic nervous systems in HF and other CVD. Moreover, NPs exert anti-hypertrophic and anti-fibrotic autocrine effects through complex signaling pathways, therefore contributing in a significant manner to cardiovascular remodeling.

NPs represent an important diagnostic and prognostic tool to be used as a biomarker in the clinical management of HF and several other cardiovascular conditions. Therapeutic strategies based on NPs’ biological properties and on their underlying molecular mechanisms have been investigated or are currently under development. The clinical applications of the discoveries related to the NP system as well as the novel therapeutic strategies developed represent an excellent example of successful translational medicine and may prompt other innovative steps towards more targeted treatment of HF and other major cardiovascular disease conditions.

## Data Availability

There are no new data associated with this article.
